# Does medial patellofemoral ligament reconstruction result in femoral tunnel enlargement? A systematic review

**DOI:** 10.1186/s43019-023-00187-1

**Published:** 2023-05-02

**Authors:** Diego Agustín Abelleyra Lastoria, Vathana Gopinath, Omkaar Divekar, Toby Smith, Tobias R. W. Roberts, Caroline B. Hing

**Affiliations:** 1 St George’s University Hospitals NHS Foundation Trust, St George’s University London, London, UK; 2 Warwick Medical School, University of Warwick, Coventry, UK; 3 Department of Trauma and Orthopaedics, Croydon University Hospital, London, UK; 4 Department of Trauma and Orthopaedics, St George’s University Hospitals NHS Foundation Trust, London, UK

**Keywords:** Medial patellofemoral ligament reconstruction, Femoral tunnel widening, Clinical outcomes, Risk factors, Systematic review

## Abstract

**Background:**

Medial patellofemoral ligament (MPFL) reconstruction is a common surgical procedure for treating patellar instability. The primary aim of this systematic review was to determine whether MPFL reconstruction (MPFLR) leads to femoral tunnel enlargement (FTE). The secondary aims were to explore the clinical effects and risk factors of FTE. Electronic databases (MEDLINE, Global Health, Embase), currently registered studies, conference proceedings and the reference lists of included studies were searched independently by three reviewers. There were no constraints based on language or publication status. Study quality assessment was conducted. 3824 records were screened in the initial search. Seven studies satisfied the inclusion criteria, evaluating 380 knees in 365 patients. Rates of FTE following MPFLR ranged from 38.7 to 77.1%. Five low quality studies reported FTE did not lead to detrimental clinical outcomes as assessed with the Tegner, Kujala, IKDC, and Lysholm scores. There is conflicting evidence regarding change in femoral tunnel width over time. Three studies (of which two had a high risk of bias) reported age, BMI, presence of trochlear dysplasia and tibial tubercle-tibial groove distance did not differ between patients with and without FTE, suggesting these are not risk factors for FTE.

**Conclusion:**

FTE is a common postoperative event following MPFLR. It does not predispose poor clinical outcomes. Current evidence lacks the ability to identify its risk factors. The reliability of any conclusions drawn is hindered by the low level of evidence of the studies included in this review. Larger prospective studies with long-term follow up are required to reliably ascertain the clinical effects of FTE.

## Background

The MPFL lies in the medial aspect of the knee, and inserts onto the upper medial aspect of the patella [1]. It contributes to knee biomechanics by drawing the patella from its lateralized position during knee extension, and drawing it towards the trochlea during knee flexion [2]. Patellofemoral instability is the perceived lack of passive control of the patella by soft tissue tethers and bony geometry [3]. It has an incidence of 5.8 per 100,000 in the adult population, with a higher rate of 29 per 100,000 in the 10 to 17 year age group [4]. The MPFL helps prevent lateral dislocation or subluxation [5]. Damage to the MPFL is likely to occur when turning or twisting the leg, predisposing to lateral patellar dislocation [6]. Though this may heal with immobilization, the ligament can become lengthened and loosened, leading to lateral patellar instability. This increases the risk of recurrent patellar dislocations in 15 to 71% of patients [7].

Medial patellofemoral ligament reconstruction is a common surgical procedure for treating patellar instability [8]. The surgical technique involves replacing the damaged ligament with a tendon graft. This is fixed to the femur by drilling a femoral tunnel, with a screw holding the graft in place [9]. A postulated complication of MPFLR is FTE [10]. However, whether FTE is a common complication of MPFLR has not been established. Change in femoral tunnel size during follow-up is seldom reported. No systematic reviews aimed at establishing FTE occurrence were identified in the literature. Therefore, the primary aim of this systematic review was to determine whether MPFLR leads to FTE.

Detrimental clinical effects of FTE have been proposed, including recurrent post-operative patellar instability [10] and low functional scores [11]. Regarding its risk factors, femoral tunnel malposition [12] and high patellar height [11] have been previously highlighted. Knowledge of risk factors for FTE could aid diagnosis via identification of high-risk groups. In addition, it could help explain the pathophysiological processes behind FTE, aiding the creation of treatment strategies. Despite clinical effects and risk factors for FTE being previously proposed, no systematic literature search aimed at summarizing these has been previously performed. Hence, the secondary aim of this review was to identify clinical effects and risk factors for FTE. We hypothesize that FTE is a common occurrence following MPFLR, re-dislocation is a potential consequence, and that initial tunnel malposition predisposes enlargement.

## Methods

The systematic review was reported in accordance with the PRISMA 2020 checklist [13].

### Study eligibility

Study eligibility was determined by following the pre-specified criteria. All in-vivo studies reporting on FTE following MPFLR were included, both full-texts and abstracts. Eligible study designs comprised case series, case–control, cross-sectional, and cohort studies, as well as randomised controlled trials. Both retrospective and prospective studies were eligible. Cadaveric studies and papers not reporting original data such as literature or systematic reviews were excluded, along with case reports, animal studies, and letters to the editor. Studies describing theoretical models were also excluded. There were no constraints based on language, publication status, patient demographics, or type of graft used. Eligibility assessment was performed by three reviewers (DAAL, VG, OD).

### Search strategy and data extraction

We searched the following electronic databases via OVID from their inception to 10/08/2022: MEDLINE, Global Health, and Embase. Currently registered studies were reviewed using the databases ISRCTN registry, the National Institute for Health Research Portfolio, the UK National Research Register Archive, the WHO International Clinical Trials Registry Platform, and OpenSIGLE (System for Information on Grey Literature in Europe). Conference proceedings from the European federation of national associations of orthopedics and traumatology (EFORT), British Orthopaedic Association and British Trauma Society were searched. The reference lists of included studies were also searched. Database search was conducted independently by three reviewers (DAAL, VG, OD). Searches were conducted twice for quality assurance. The search strategy is presented in Appendix 1.

### Methodological appraisal

Level of evidence and risk of bias of included studies were evaluated independently by two reviewers (DAAL, OD). The level of evidence of the studies presented was determined with the March 2009 Oxford Centre for Evidence-Based Medicine: Levels of Evidence [14]. The Institute of Health Economics case series studies quality appraisal checklist was used to determine risk of bias of case series [15]. The Cochrane Collaboration’s Risk of Bias in Non-Randomized Studies—of Interventions tool was used to perform a risk of bias assessment for non-randomised studies [16].

### Data analysis

Quantitative pooled analysis was prevented by the heterogeneity of the data in terms of criteria for FTE, approach to MPFLR, and methods of assessment of clinical outcomes. Therefore, a narrative synthesis was performed. The effects of MPFLR on femoral tunnel width were evaluated. Baseline characteristics including number of patients, number of knees, patient sex, age, follow-up duration, and imaging method were extracted (Table 1). Rates of FTE, its clinical effects (measured with validated outcome scores) and predisposing factors were summarized in Table 2 and evaluated. A parameter was deemed a risk factor for FTE when there was a statistically significant correlation between both, or when the parameter was significantly different in patients with and without FTE (Table 3).

**Table 1:** Baseline characteristics of studies included

Study	Level of evidence (study design), risk of bias	Graft used	Type of patellar instability	Number of patients (males, females)	Number of knees	Patient age (years)	Follow-up duration	Imaging method
Turgay et al., 2017	4 (case series), NFT	Gracilis tendon graft (whether auto- or allograph not reported)	NR	31 (8, 23)	31	Not reported	4.1 years	MRI
Schüttler et al., 2018	4 (case series), high	Gracilis tendon autograft	NR	49	51	22.6	3 years	MRI
Berard et al., 2013	3 (case control), low	Gracilis tendon autograft	Episodic patellar dislocations	51 (14, 37)	55	24.2	3.7 years	Lateral X-ray
Neri et al., 2019	4 (case series), high	Gracilis tendon graft (whether auto- or allograph not reported)	Recurrent dislocations (> 2)	107	112	25	4.9 years	3D CT
Kita et al., 2017	4 (case series), high	Semitendinosus tendon autograft	Recurrent dislocations	23 (6, 17)	23	24	2 years	3D CT
Wong et al., 2021	4 (case series), some concerns	30 allographs (18 semitendinosus, 12 gracilis), 6 autographs (3 semitendinosus, 1 quadriceps, 2 gracilis)	16 had recurrent dislocations. Type of instability not reported in remaining 22	38 (16, 22)	38	20.4	2.5 years	MRI
Qin et al., 2017	4 (case series), some concerns	Gracilis tendon autograft	Recurrent dislocations	66 (18, 48)	70	24.3	20.9 months	CT

*NR* not reported, *NFT* non-full text article, *MRI* magnetic resonance imaging, *3D CT* three-dimensional computerize tomography

**Table 2:** The occurrence and clinical effects of FTE following MPFLR

Study	Criteria for FTE	FTE	Clinical effect of FTE
Turgay et al., 2017	Not reported	12 (38.7%) patients experienced femoral tunnel enlargement, and 75% of these patients had proximal tunnel misplacement. Of the 19 (61.3%) patients without femoral tunnel expansion, only 32% had proximal tunnel misplacement	There were no differences in Tegner, Kujala, and IKDC scores in patients with and without femoral tunnel enlargement
Schüttler et al., 2018	Increase to more than double of the femoral tunnel’s original area in at least two of its sections	A total of 23 (45.1%) knees experienced FTE	Patients with FTE displayed significantly better outcomes in terms of symptoms and performance of daily activities according to the Kujala (84 vs. 75, *p* = 0.032) and IKDC (80 vs. 71, *p* = 0.024) scores, but not as measured with the Tegner score (4.2 vs 3.9)
Berard et al., 2013	Increase to more than double of the femoral tunnel’s original area	Of the 55 patients, 23 (41.8%) experienced FTE The mean cross-sectional area of the tunnel in the non-enlarged group was 57.9 mm2, compared with 105 mm2 in the enlarged group (P < .001)	IKDC scores did not differ between patients with and without FTE (82.6 vs 83.0, respectively, *p* = 0.93). There was no difference in risk of patellar instability between both groups (*p* = 1.0)
Neri et al., 2019	Percentage increase in tunnel cross-sectional area from baseline was calculated	Number of patients experiencing FTE were not reported. Degree of FTE was not reported. Malposition of the femoral tunnel was correlated with FTE	FTE predicted lower functional scores. Increases in femoral tunnel area at 5 mm, 15 mm, and 25 mm from the medial femoral cortex were negatively associated with post-operative Kujala and IKDC scores (− 0.535 and − 0.557, − 0.331 and − 0.296, − 0.218 and − 0.193, respectively)
Kita et al., 2017	Percentage increase in the aperture and inside the tunnel cross-sectional area from baseline was calculated	Cross sectional area at the aperture increased from 21.7 mm^2^ at 3 weeks to 30.3 mm^2^ 1-year post-op (41.1% increase, *p* > 0.05). Area 5 mm from the aperture increased from 21.9 to 23.8 mm^2^ (8.8% increase, *p* > 0.05), and area 10 mm from the aperture increased from 22.1 to 22.7 mm^2^ (2.6% increase, *p* > 0.05)	Femoral tunnel enlargement was not associated with post-operative Kujala scores (*r* = − 0.015, *p* = 0.946)
Wong et al., 2021	Calculated by subtracting the size of the screw used from the measured opening tunnel size	Mean increase in femoral tunnel size was 2.5 mm	Not reported
Qin et al., 2017	Increase in femoral tunnel width at 3 days and last follow-up was measured	At the last follow-up, there were 54 knees with tunnel enlargement (77.1%), and 16 without (22.9%). Femoral tunnel width was 8.7 mm at 3 days, and 10.6 mm at last follow-up. There was a 21.8% increase (*p* < 0.05)	Average Kujala score was 82.5 in patients with FTE, compared to 79.4 in those without (*p* = 0.386). Lysholm score was 84.8 in patients with FTE, compared to 78.6 in those without (*p* = 0.085)

*FTE* femoral tunnel enlargement, *IKDC* international knee documentation committee

**Table 3:** Comparison of studies stratifying outcomes according to presence of FTE

Parameter	Berard et al., 2013			Schüttler et al., 2018		
Group	FTE (*n* = 23)	Non-FTE (*n* = 32)	*P*-value	FTE (*n* = 23)	Non-FTE (*n* = 28)	P-value
Sex distribution (males, females)	NR	NR	NR	11, 12	6, 22	NS
BMI (kg/m^2^)	23.2	21.8	0.12	24.2	25.2	NS
Age (years)	24.4	24.5	0.97	20.6	24.3	NS
% with no trochlear dysplasia	4.3	6.2	0.14	4.3	21.4	NS
Presence of patella alta	52.2	28.2	0.09	NR	NR	NR
C-D index	1.17	1.08	0.03	1.0	1.0	NS
% Femoral tunnel malpositioned	43.5	34.4	0.58	AP: 26 PD: 87	AP: 32 PD: 46	AP: NS PD: 0.0033
% with Increased TT-TG distance (> 20 mm)	8.7	9.4	0.69	8.7	14.3	NS
TT-TG distance (mm)	16.2	15.4	0.57	14.8	13.0	NS
Patients with recurrent subluxation/dislocation	1	2	1.0	1	0	NS
Knee flexion	NR	NR	NR	137°	136°	NS
% with cartilage damage	NR	NR	NR	39%	25%	NS
Patients with positive apprehension test post-op	NR	NR	NR	3	1	NS

*FTE* femoral tunnel enlargement, *BMI* body mass index, *NS* not statistically significant, *NR* not reported, *C-D* Caton-Deschamps, *AP* anterior posterior, *PD* proximal–distal, *TT–TG* tibial tubercle–tibial groove

## Results:

**Fig. 1:**
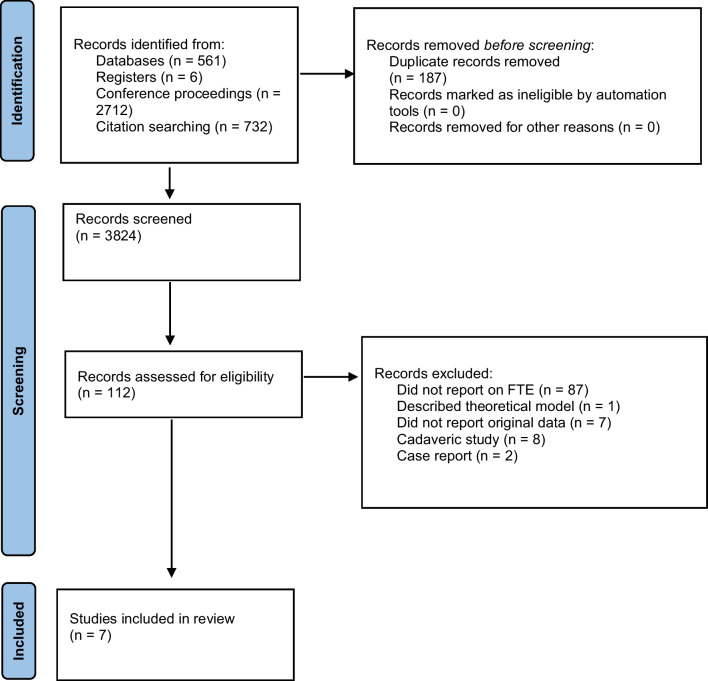
PRISMA diagram depicting the study collection process

A total of 3824 records were screened, with 112 potentially eligible articles identified (Fig. 1). A total of 105 were excluded on the bases of the pre-specified exclusion criteria. Seven studies were included, evaluating 380 knees of 365 patients. Mean patient age ranged from 20.4 to 25 years. Of the 380 grafts used, 333 were gracilis tendon grafts (178 autographs, 12 allographs, 143 with origin not reported). Of the 21 semitendinosus grafts used, 18 were allografts, three were autografts. A single quadriceps tendon autograft was used. Three studies utilized bioabsorbable screws [10, 17, 18]. Qin et al. used both titanium and bioabsorbable screws [19], whereas type of screw used was not reported in three studies [11, 12, 20]. Of the 365 patients identified, 212 had recurrent patellar dislocation (more than two previous episodes). Berard et al. reported on 51 patients with episodic patellar dislocations, but did not specify which frequency this entailed [10]. Type of patellar instability was not reported in 102 patients. All studies diagnosed FTE when its width or cross-sectional area increased from baseline.

### Study quality assessment

The findings of the study quality assessment are presented in Table 1. Of the seven studies included, six were case series. These carried a low-level of evidence of four. Risk of bias could not be assessed in one case series due to this not being a full-text study [12]. Risk of bias was deemed high in three case series due to missing data [11, 17, 20]. Schüttler et al. did not report *p*-values of non-statistically significant differences [17]. Neri et al. did not report degree of FTE [11], whereas Kita et al. reported association between FTE and potential risk factors without reporting values for these [20]. The case series by Qin et al. [19] and Wong et al. [18] carried some concerns regarding their risk bias due to their retrospective nature and being performed in a single center. Only one non-randomised comparative study was identified [10]. This presented with a level of evidence three and low risk of bias. Overall, the majority of studies included in this review exhibited methodological limitations in terms of study design and risk of bias (Table 1).

### Occurrence femoral tunnel enlargement

A total of seven studies reporting on femoral tunnel width following MPFLR were identified (Table 2). Criteria for FTE was not reported in one study [12]. Berard et al. [10] and Schüttler et al. [17] diagnosed FTE when the femoral tunnel’s original surface area increased to twice its original size. Kita et al. [20] and Neri et al. [11] measured percentage increase in femoral tunnel area from baseline, while Qin et al. [19] and Wong et al. [18] reported absolute increase in femoral tunnel surface area. Rates of FTE ranged from 38.7 to 77.1%.

### Clinical effects of femoral tunnel enlargement

Of the five studies reporting on the consequences of FTE, four reported that this did not lead to negative clinical outcomes [10, 12, 19, 20]. Schüttler et al. [17] reported better outcomes in patients with FTE, whereas Neri et al. [11] observed FTE predicted lower functional scores.

Turgay et al. [12] reported no differences in Tegner Activity Scale, Kujala Patellofemoral Disorder Score, and the International Knee Documentation Committee (IKDC) scores in patients with and without FTE. Neither scores nor *p*-values were reported [12]. Berard et al. found IKDC scores did not differ between patients with and without FTE (82.6 vs 83.0, respectively, *p* = 0.93) [10]. There was a single case of recurrent subluxation reported in the FTE group (4.3%). There was a case of subluxation (6.2%) and one case of dislocation (3.1%) in the non-FTE group. There was no difference in risk of patellar instability between patients with and without FTE (*p* = 1.0) [10].

Kita et al. found FTE was not associated with post-operative Kujala scores (*r* = − 0.015, *p* = 0.946) [20]. Moreover, Qin et al. reported that average Kujala score was 82.5 in patients with FTE, compared to 79.4 in those without (*p* = 0.386). Lysholm score was 84.8 in patients with FTE, compared to 78.6 in those without (*p* = 0.085) [19].

Schüttler et al. reported better outcomes in terms of symptoms and performance of daily activities in patients with FTE than in those without [17]. Patients with FTE displayed significantly better outcomes in terms of symptoms and performance of daily activities according to the Kujala (84 vs. 75, *p* = 0.032) and IKDC (80 vs. 71, *p* = 0.024) scores, but not as measured with the Tegner score (4.2 vs 3.9, *p* > 0.05) [17]. In contrast, Neri et al. observed FTE predicted lower functional scores [11]. Increases in femoral tunnel area at 5 mm, 15 mm, and 25 mm from the medial femoral cortex were negatively associated with post-operative Kujala and IKDC scores (− 0.535 and − 0.557, − 0.331 and − 0.296, − 0.218 and − 0.193, respectively) [11].

### Femoral tunnel enlargement according to tracking period

All included studies reported duration of follow-up for the assessment of clinical outcomes. However, three studies did not report the point at follow-up in which femoral tunnel width was measured [12, 17, 18]. Two studies measured femoral tunnel width at only one point during follow-up. Neri et al. [11] measured it at six months post-operatively, whereas Berard et al. [10] reviewed it at 12 months. Two studies assessed femoral tunnel width at two time-points during follow-up [11, 20]. Kita et al. found cross-sectional area of the femoral tunnel aperture increased from 21.7 mm^2^ at three weeks to 30.3 mm^2^ 12 months post-operatively (41.1% increase, *p* > 0.05) [20]. Cross sectional area 5 mm from the aperture increased from 21.9 to 23.8 mm^2^ (8.8% increase, *p* > 0.05), and the area 10 mm from the aperture increased from 22.1 to 22.7 mm^2^ (2.6% increase, *p* > 0.05) [20]. Qin et al. found the average femoral tunnel width was 8.7 mm at 3 days, and 10.6 mm at last follow-up (time elapsed from operation was unspecified). There was a 21.8% increase (*p* < 0.05) [19].

### Risk factors of FTE

Only two studies reported outcomes for patients with and without FTE separately [10, 17]. Their findings are compared in Table 3. Schüttler et al. found a significantly higher rate of proximal tunnel malposition in patients with FTE (87%, compared to 46% of knees with no FTE, *p* < 0.01) [17]. Antero-posterior malposition was observed in 26% of knees with FTE, compared to 32% in those without (*p* > 0.05). Malposition was diagnosed when the femoral tunnel aperture was located > 7 mm away from Schöttle’s [21] point. There was no correlation between the amount of malposition and the amount of FTE [17]. In addition, Schüttler found no differences between FTE and non-FTE knees in terms of patellar height, age, body mass index (BMI), cartilage damage, trochlear dysplasia, and tibial tubercle-trochlear grove (TT-TG) distance. *P*-values for these differences were not reported [17]. Berard et al. [10] contradicted Schüttler’s [17] findings in that increased patellar height was associated with an increased risk of FTE (*p* = 0.03). In addition, there was no correlation between femoral tunnel malposition and FTE (*p* = 0.58). Malposition was diagnosed when femoral tunnel aperture was located > 7 mm away from Schöttle’s point [10].

The lack of a statistically significant association between femoral tunnel position and FTE was also observed by Kita et al. [20]. They also found FTE was not correlated with age (*p* = 0.41), BMI (*p* = 0.28), Insall-Salvati ratio (*p* = 0.37), sulcus angle (*p* = 0.76), congruence angle (*p* = 0.58), lateral tilt angle (*p* = 0.55), TT-TG distance (*p* = 0.12), presence of trochlear dysplasia (*p* = 0.92), and antero-posterior and proximal–distal position of the femoral tunnel center (*p* = 0.38 and *p* = 0.87, respectively) [20]. Values for these parameters were not reported. Distance from the anterior border to the posterior border of the femoral condyle was defined as 100%. Antero-posterior and proximal–distal positions of the femoral tunnel center were calculated relative to this distance [20].

## Discussion

As hypothesized, FTE commonly occurs following MPFLR. Rates of FTE ranged from 38.7 to 77.1% in the studies identified. Though current evidence suggests FTE does not lead to poor clinical outcomes, it lacks the ability to identify its risk factors. Five studies found patients with FTE did not exhibit worse outcome scores than those without [10–12, 17, 20]. The concordance between multiple studies’ findings strengthens the claim that FTE does not lead to detrimental clinical outcomes, contradicting our initial hypothesis. However, they included 365 patients, which may not be sufficiently powered to confidently ascertain the clinical effects of FTE. In addition, they carried a low level of evidence and concerns regarding their risk of bias, which hinder the validity of their findings. Further research should report on FTE and its effects on clinical outcomes given the lack of literature on the subject. Cregar et al. conducted a systematic review of risk factors for MPFLR failure [22]. They found that FTE predisposed negative clinical outcomes. However, their conclusion is severely limited by the inclusion of a single study evaluating this parameter. This was a study carried out by Neri et al. [11] which was also included in this review. However, its findings are outweighed by five studies reporting no link between FTE and worsened clinical outcomes.

The point during the post-operative period in which FTE occurs remains unclear. Three studies did not report the point at follow-up in which femoral tunnel width was measured [12, 17, 19]. In addition, two studies measured femoral tunnel width at only one point during follow-up [10, 11]. Only two studies reported on femoral tunnel width at two different time-points during follow-up. Kita et al. [20] measured it at three weeks and 12 months post-operatively, whereas Qin et al. [19] did so at three days post-operatively and at a later unspecified point. Kita et al. [20] found no statistically significant increase in cross-sectional area. Qin et al. found average femoral width increased significantly [19]. Differing findings means it is not possible to determine whether femoral tunnel width increases with time following MPFLR. Further research should report on femoral tunnel width at multiple points during follow-up to ascertain when FTE occurs.

Regarding predisposing factors for developing FTE, three studies found that age, BMI, presence of trochlear dysplasia, and TT-TG distance did not differ between patients with and without FTE [10, 17, 20]. The concordance between three studies’ findings strengthens the claim these may not be risk factors for developing FTE. However, these disagreed regarding the effect of femoral tunnel position on femoral tunnel size. Berard et al. [10] and Kita et al. [20] found these were not correlated, whereas Schüttler et al. [17] did. This could be attributed to differences in criteria for diagnosing femoral tunnel malposition and FTE. Therefore, whether these are correlated remains unclear. In addition, FTE occurred regardless of whether bioabsorbable or metal screws were used [10, 17–19]. This has also been observed in relation to anterior cruciate ligament reconstruction (ACLR) [23]. However, a titanium screw was used in one study only [19]. Further studies using metal screws during MPFLR should report on FTE to determine whether these are associated. There is a discrepancy in current evidence regarding the effect of patellar height on FTE [10, 17, 20]. In addition, an association between FTE and parameters explored in a single study cannot be reliably established. These were patient sex [17], sulcus angle, congruence angle and lateral tilt angle [20]. Therefore, further research evaluating these parameters is required to ascertain whether they are risk factors for FTE.

Femoral tunnel enlargement has been widely studied in relation to ACLR [24–26]. This uses techniques similar to MPFLR to create and utilize the femoral tunnel [17, 27]. Risk factors for FTE and malposition may be similar in ACLR and MPFLR. Ligamentisation following ACLR is the conversion of the tendon autograft into a ligament similar to the native ACL in both biochemical and histological criteria. This leads to graft swelling [28]. Swelling of the graft could apply pressure to the aperture of the femoral tunnel, increasing its diameter. It was conjectured by Qin et al. that a graft used in MPFLR could undergo ligamentisation in the first-year post-intervention [19]. This graft could then lack the necessary strength to hold the patella, giving rise to graft tunnel motion, and, as a result, FTE. However, since this is hypothetical and has not been studied in relation to MPFL reconstruction, it necessitates evaluation.

To avoid FTE, Kita et al. recommend immobilizing the knee after surgery, since weight bearing can lead to FTE [20]. Applying weight on the knee early after surgery can result in dynamic knee valgus and hip internal rotation, which can put pressure on the femoral tunnel graft and amplify tunnel enlargement [29]. However, the relationship between post-operative mobilization and FTE was not explored in any of the studies included in this review. Therefore, there is no evidence to suggest that current practice of early mobilization following knee surgery should be altered, particularly as this aids return to pre-operative activity levels [30]. Further research should evaluate the impact of early mobilization on FTE.

Our study methodology was strengthened by searching multiple literature sources, including electronic databases, conference proceedings, currently registered studies, and the reference lists of studies included. Search was conducted independently by three reviewers, at two different time points for quality assurance. This minimized the risk of missing potentially relevant studies. This review expands the knowledge of clinicians, in that femoral tunnel enlargement does not predispose poor clinical outcomes, preventing unnecessary revision surgery. In addition, it highlights factors which do not predispose FTE, guiding MPFLR post-operative management. Identifying risk factors for FTE and its clinical effects remains challenging due to limitations in current evidence. Firstly, the studies included in this review have differing methodologies, such as approach to MPFLR (e.g., differing techniques and grafts) and therefore it was not possible to perform quantitative pooled analysis. Secondly, criteria for diagnosing FTE varied between studies, leading to the adequacy of femoral tunnel size being interpreted differently by different authors. This led to the wide variability in rates of FTE between studies. Two studies diagnosed FTE when it reached an area twice its original size [10, 17], and therefore could have missed slight widening. Further research should aim to determine the femoral tunnel size that leads to detrimental clinical outcomes, and adopt a standardized definition of FTE. Thirdly, no studies reported results according to participants’ ethnicity. Considering this affects joint hypermobility [31], further studies should stratify outcomes according to ethnicity to determine whether it impacts prognosis following MPFLR. Fourthly, level of evidence was low, with no prospective cohort studies comparing outcomes in patients with and without FTE identified. Most studies were retrospective, which introduces a potential risk of bias. The major methodological limitations of the studies included in this review hinder the validity of any conclusions drawn. Further high-quality prospective cohort studies are required to ascertain the clinical effects of FTE. Fifthly, the effect of FTE following MPFLR in teenagers was not explored. This should be evaluated considering the high incidence of patellar dislocation in this age group, estimated at 29 per 100,000 for 10 to 17-year olds [4]. Finally, study screening process rendered the exclusion of 87 studies not reporting on femoral tunnel size following MPFLR (Fig. 1). Better understanding of its effects on clinical outcomes is unlikely unless this parameter is further explored. Femoral tunnel size following MPFLR cannot be used as a single prognostic factor in patients undergoing MPFLR. The etiology of negative outcomes following MPFLR is multifactorial, with graft tension, patella location, underlying trochlear dysplasia and ligamentous hyperlaxity playing a role [9]. In summary, limitations of current evidence include differing approaches to MPFLR and criteria to diagnose FTE (preventing pooled analysis), lack of outcome stratification according to ethnicity and age, and low level of evidence.

## Conclusion

Femoral tunnel enlargement is a common postoperative event following MPFLR. It does not predispose poor clinical outcomes. Current evidence lacks the ability to identify its risk factors. The reliability of any conclusions drawn is hindered by the low level of evidence of the studies included in this review. Larger prospective studies with long-term follow up are required to reliably ascertain the clinical effects of FTE.

## Data Availability

Not applicable.

## Appendix 1: Search strategy

1. (MPFL OR Medial patellofemoral ligament)
2. (repair OR surgery OR reconstruction OR operation OR intervention OR procedure)
3. 1 AND 2
4. MPFLR
5. 3 OR 4
6. Bone tunnel OR femoral tunnel OR tunnel OR drill*
7. Widen* OR enlarge* OR size OR diameter OR surface area OR area OR expan* OR broad* OR cross sectional area
8. 5 AND 6 AND 7

Deduplicate

## Abbreviations

MPFL: Medial patellofemoral ligament
MPFLR: Medial patellofemoral ligament reconstruction
FTE: Femoral tunnel enlargement
TT-TG: Tibial tubercle-tibial groove
MCL: Medial collateral ligament
IKDC: International Knee Documentation Committee
ACLR: Anterior cruciate ligament reconstruction
